# Antibody levels versus vaccination status in the outcome of older adults with COVID-19

**DOI:** 10.1172/jci.insight.183913

**Published:** 2024-10-22

**Authors:** Sylvia Mink, Christoph H. Saely, Andreas Leiherer, Patrick Reimann, Matthias Frick, Janne Cadamuro, Wolfgang Hitzl, Heinz Drexel, Peter Fraunberger

**Affiliations:** 1 Central Medical Laboratories, Feldkirch, Austria.; 2 Private University in the Principality of Liechtenstein, Triesen, Principality of Liechtenstein.; 3 VIVIT Institute and; 4 Department of Internal Medicine, Academic Teaching Hospital Feldkirch, Feldkirch, Austria.; 5 Department of Laboratory Medicine, Paracelsus Medical University Salzburg, Salzburg, Austria.; 6 Department of Research and Innovation, Team Biostatistics and Publication of Clinical Trials, Paracelsus, Medical University, Salzburg, Austria.; 7 Drexel University College of Medicine, Philadelphia, Pennsylvania, USA.

**Keywords:** Infectious disease, Vaccines, Clinical practice

## Abstract

**BACKGROUND:**

Despite the currently prevailing, milder Omicron variant of COVID-19, older adults remain at elevated risk of hospital admission, critical illness, and death. Loss of efficacy of the immune system, including reduced strength, quality, and durability of antibody responses, may render generalized recommendations on booster vaccinations inadequate. There is a lack of data on the efficacy of antibody levels in older adults and on the utility of vaccination status versus antibody levels as a correlate of protection. It is further unclear whether antibody levels may be used to guide the timing of booster vaccinations in older adults.

**METHODS:**

We conducted a prospective multicenter cohort study comprising hospitalized patients with COVID-19. Anti–SARS-CoV-2 spike antibodies were measured on hospital admission. The primary endpoint was in-hospital mortality. Patients were stratified by age, antibody levels, and vaccination status. Multiple logistic regression and Cox regression analyses were conducted.

**RESULTS:**

In total, 785 older patients (≥60 years of age [a]) and 367 controls (<60a) were included. After adjusting for confounders, risk of mortality, ICU admission, endotracheal intubation, and oxygen administration was 4.9, 2.6, 6.5, and 2.3 times higher, respectively, if antibody levels were < 1,200 BAU/mL (aOR, 4.92 [95%CI, 2.59–9.34], *P* < 0.0001; aOR, 2.64 [95%CI, 1.52–4.62], *P* = 0.0006; aOR, 6.50 [95%CI, 1.48–28.47], *P* = 0.013; aOR, 2.34 [95%CI, 1.60–3.343], *P* < 0.0001). Older adults infected with the Omicron variant were approximately 6 times more likely to die if antibody levels were < 1,200 BAU/mL (aOR, 6.3 [95% CI, 2.43–16.40], *P* = 0.0002).

**CONCLUSION:**

Antibody levels were a stronger predictor of in-hospital mortality than vaccination status. Monitoring antibody levels may constitute a better and more direct approach for safeguarding older adults from adverse COVID-19 outcomes.

## Abstract 2

Anti-SARS-CoV-2 spike antibody levels were a stronger predictor of in-hospital mortality than vaccination status in older adults with COVID-19. Older adults with antibody levels &lt;1200BAU/ml should be considered for additional booster vaccinations.

## Introduction

To date, the World Health Organization (WHO) has recorded over 775 million confirmed cases of COVID-19 worldwide (1). However, following the WHO’s declaration of the official end to the COVID-19 public health emergency of international concern in May 2023 (2), testing for SARS-CoV-2 infections significantly decreased, and detailed reporting of infection numbers ceased (3).

Nonetheless, new SARS-CoV-2 variants keep being identified (1), such as the latest variant of interest, JN.1, a descendant of BA.2.86 that exhibits an additional mutation in the spike protein. The variant’s prevalence has been rapidly increasing in the Americas, the Western Pacific, and the European regions, and it currently constitutes the overwhelming majority of BA.2.86 descendent lineages (1). With the virus persisting in the human population and new variants of interest being reported (4), SARS-CoV-2 is going to continue to affect vulnerable groups in particular.

Older adults are particularly susceptible to COVID-19, with age being an independent risk factor for hospital admission, critical illness, and death (5, 6). The infection fatality rate of COVID-19 increases up to 4 times every 10 years (7). In the United States, in the period of 2020 to 2023, deaths involving COVID-19 accounted for approximately 10% of total deaths in adults aged 50 years or older (8). Another study reported a 3- to 4-fold increase in COVID-19 infection fatality rates with every 20 years of age (9). In addition, weekly COVID-19 mortality rates showed a direct correlation with the proportion of individuals aged 65 years or older within a population (10). Older adults thus continue to be in need of protective measures such as regular booster vaccinations (11).

An accumulation of comorbidities (12), general frailty (11), and the diminished function of both the innate and the adaptive immune system have been suggested to put older adults at risk (13–16). Factors that affect the innate immune system in older adults include a reduced type I IFN response (13), a decline in angiotensin converting enzyme 2 (ACE2) expression that is associated with heightened proinflammatory responses (14), and decreased phagocytosis efficiency and NK cell function (15) as well as chronic low-grade inflammation (16).

Regarding the adaptive immune system, an accumulation of aberrant, age-associated B cells, reduced T cell function, and a decline in humoral immune responses have been described (17, 18). Age-related bone marrow degeneration results in decreased production of naive B lymphocytes (19). Although peripheral plasma cell counts may remain unaffected, the majority of these cells has previously had contact to an antigen, which limits the ability to bind new antigens. These factors decrease and delay the production of antibodies against new epitopes (14).

In addition, antibody levels diminish at a faster rate in older adults, and antibody affinity decreases with age (13, 20, 21) due to reduced somatic hypermutation, decreased rates of isotype switching, and lower rates of spontaneous mutations in variable regions (22). Furthermore, responses to vaccines differ widely between individuals and generally decline with age, with older adults exhibiting poor antibody production and reduced durability of vaccine-induced immune response (18, 23).

With progressing age, the prevalence of certain comorbidities that are associated with reduced antibody responses also increases. For instance, the prevalence of chronic kidney disease (CKD) is known to rise from 13.7% in patients aged 30–40 years to 27.9% in patients aged 70–80 years (24, 25). A combination of several factors, such as uremia, heightened intestinal permeability, increased oxidative stress, and elevated levels of proinflammatory cytokines are thought to contribute to low-grade systemic inflammation and premature aging of the immune system, resulting in reduced vaccine efficacy (26). In addition, comorbidities such as type 2 diabetes (T2D), rheumatoid arthritis, chronic pulmonary diseases, and haemato-oncological diseases were also associated with reduced antibody responses to COVID-19 vaccines (27–30).

Generalized recommendations regarding the timing and frequency of booster vaccinations may, therefore, be inadequate to protect older adults. There are currently no clear indicators of when older adults are sufficiently protected. Neither is there an instrument available to guide recommendations for vaccination and booster strategies in older adults.

While anti–SARS-CoV-2 antibody levels have been linked to outcome in COVID-19 (31), no protective threshold has been defined in older adults. Such a protective threshold could aid clinicians in recommending timely booster vaccinations to help protect vulnerable patients from severe courses and elevated COVID-19–related mortality (32).

There is a lack of data on the efficacy of antibody levels in this important patient subset, and, to our knowledge, no comparisons on the utility of vaccination status versus antibody levels for predicting severe courses, including COVID-19–related mortality, have been conducted in older adults (33).

In this prospective, multicenter cohort study, we therefore evaluated how antibody levels are associated with outcome in older adults with COVID-19, who remain at high risk of severe courses and mortality. We further aimed to determine the risk of adverse outcomes in relation to antibody levels to help guide recommendations for when booster vaccinations in older adults should be considered. Finally, we evaluate the utility of antibody levels versus vaccination status as a correlate of protection against adverse outcomes including COVID-19 mortality in hospitalized older adults with COVID-19.

## Results

### Participants.

**Figure 1:**
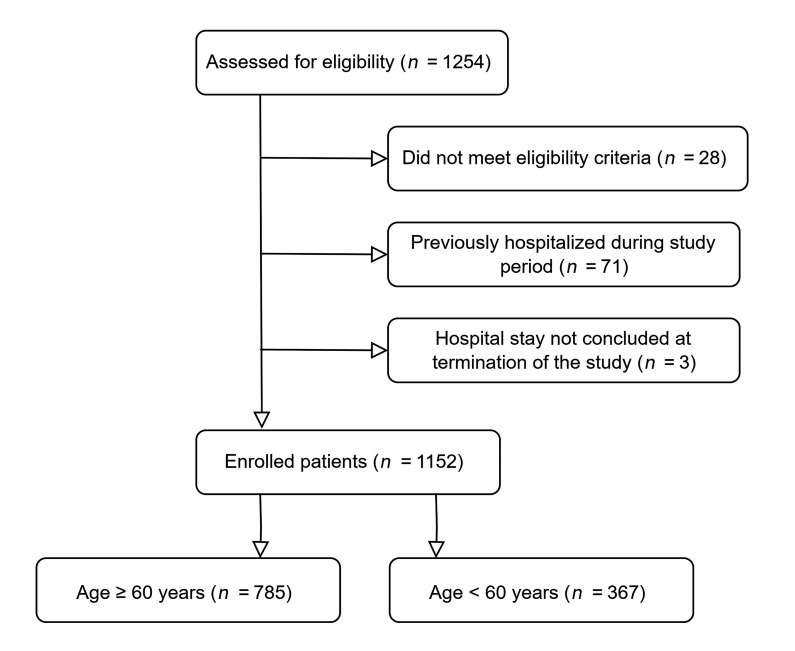
Patient flow diagram.

During the period spanning from August 1, 2021, and April 10, 2022, a total of 1,254 hospitalized patients with COVID-19 from 5 study centers were evaluated for eligibility. Out of this initial group, 1,152 patients were included in the study. In total, 785 patients were aged 60 years or above and thus classified as older adults. The control group encompassed 367 adults younger than 60 years. Patient flow is depicted in Figure 1.

With regard to the whole cohort, 118 patients died, 165 were admitted to an intensive care unit, 47 patients required endotracheal intubation for respiratory support, and 587 patients required oxygen administration. The majority of these cases concerned older adults, who accounted for 112 deaths, 108 ICU admissions, 33 intubations, and 450 instances of oxygen administration. Median duration of hospital stay was 8 days (interquartile range [IQR], 4–16) overall and 10 days (IQR, 5–18) in older adults. Patient characteristics of older and younger adults are outlined in Table 1, Table 2, and Table 3.

### Outcome by antibody levels.

Anti–SARS-CoV-2 antibodies were significantly lower in older adults who died compared with those who survived (mean 408 binding antibody units [BAU]/mL [95% CI, 242–574], versus mean 1,146 BAU/mL [95% CI, 1,057–1,236]; *P* < 0.0001).

Older adults whose antibody levels fell below the threshold of 1,200 BAU/mL were more than 4 times more likely to die compared with those with antibody levels above this threshold (OR, 4.41 [95% CI, 2.57–7.56]; *P* < 0.0001).

With regard to secondary endpoints, older adults who required oxygen administration, endotracheal intubation, or intensive care treatment also exhibited significantly lower antibodies compared with patients who did not require these interventions (oxygen administration: mean 787 BAU/mL [95% CI, 686–889] versus 1,377 BAU/mL [95% CI, 1,249–1,504], *P* < 0.0001; endotracheal intubation: mean 204 BAU/mL [95% CI, 0–429] versus 1,075 BAU/mL [95% CI, 991–1,159], *P* < 0.0001; intensive care: mean 587 BAU/mL [95% CI, 391–783] versus 1,111 BAU/mL [95% CI, 1,022–1,200], *P* < 0.0001).

**Figure 2:**
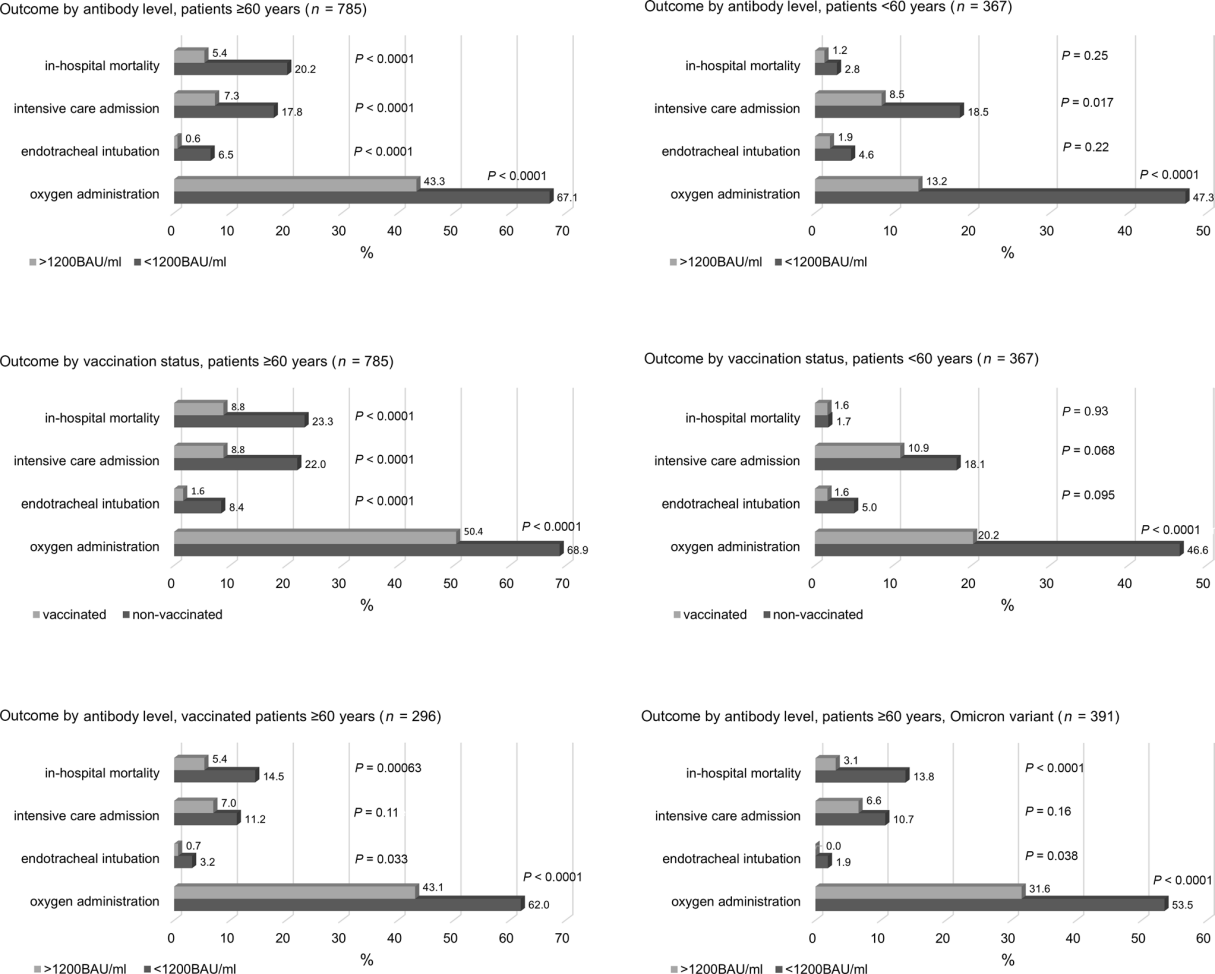
Patient outcomes for older (≥60 years) and younger adults (<60 years) in percentages regarding in-hospital mortality, intensive care treatment, endotracheal intubation, and oxygen administration by antibody level and vaccination status. Bottom row: Outcome by antibody level in vaccinated older adults and older adults infected with the Omicron variant. BAU binding antibody units.

Figure 2 depicts patient outcomes in percentages with regard to the endpoints all-cause in-hospital mortality, ICU treatment, endotracheal intubation, and oxygen administration by antibody level and vaccination status.

Vaccinated older adults had lower rates of in-hospital mortality, ICU admission, endotracheal intubation, and oxygen administration compared with nonvaccinated patients but higher rates than patients with antibody levels above 1,200 BAU/mL.

### Survival over time.

**Figure 3:**
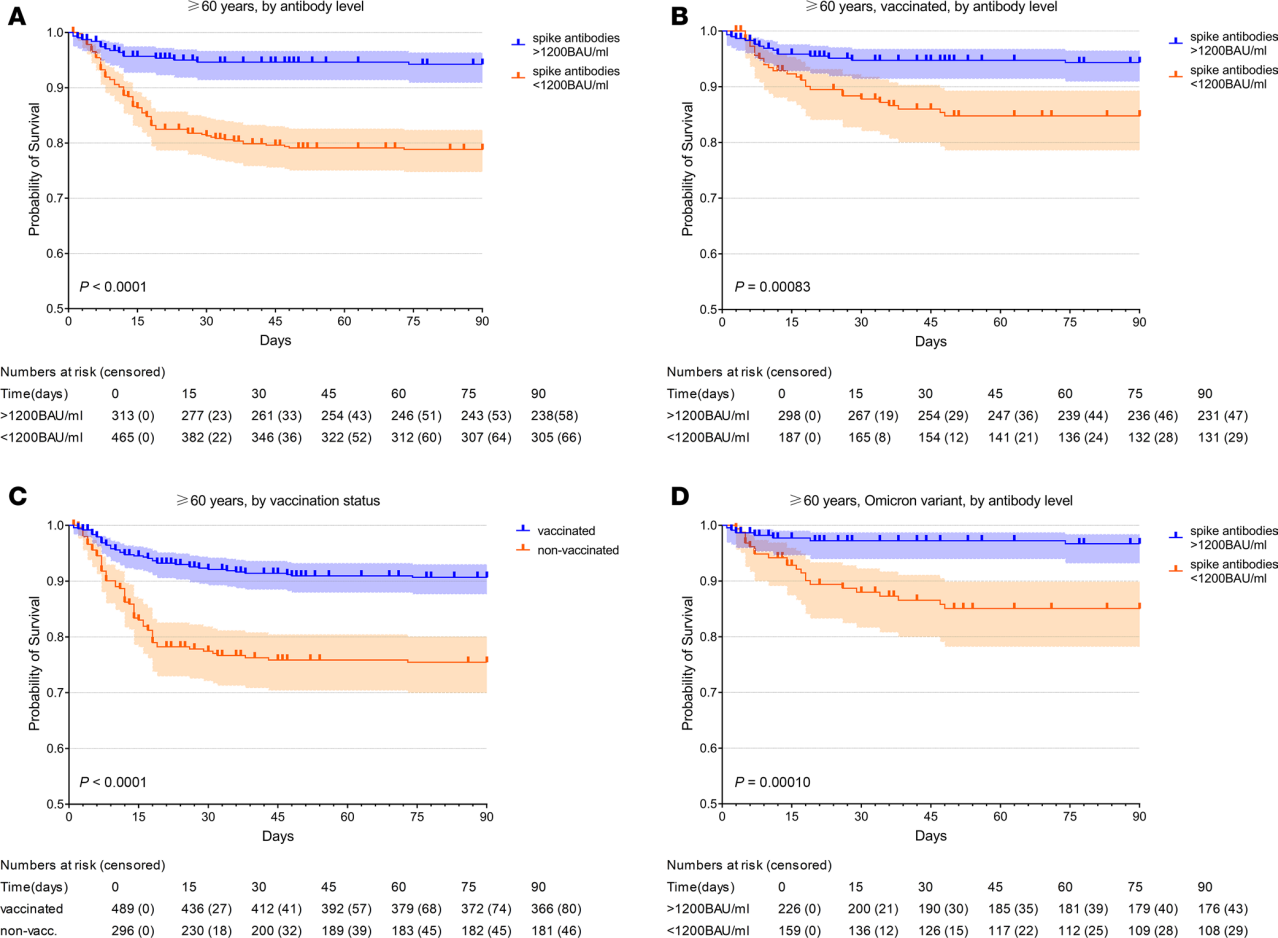
Cumulative survival over time in older adults. (**A**–**D**) Kaplan-Meier curves with 95% CI, for cumulative survival over time in older adults (≥60 years) by high and low anti–SARS-CoV-2 spike antibody level (above and below 1,200 BAU/mL) (**A**), by vaccination status (**B**), by antibody level in vaccinated older adults (**C**), and by antibody level in older adults infected with the Omicron variant (**D**). Number censored: cumulative number of patients lost to follow-up. Statistical significance was determined by log rank (Mantel-Cox) test. BAU, binding antibody units; nonvacc., nonvaccinated patients.

Figure 3 shows Kaplan-Meier curves for cumulative survival over time by antibody level for older adults, vaccinated older adults, and older adults infected with the currently prevailing Omicron variant. For comparison, cumulative survival of older adults by vaccination status is also included. Statistical significance was tested by log rank (Mantel Cox) test. Median follow-up time was 90 days after hospital admission (IQR, 48–90 days.)

In older adults, vaccinated patients had better cumulative survival than nonvaccinated patients but had lower cumulative survival than those with antibodies above 1,200 BAU/mL.

While both vaccination status and antibodies above 1m200 BAU/mL are good predictors of protection from in-hospital mortality, patients with spike antibodies above 1,200 BAU/mL had better odds of survival than vaccinated patients (OR, 4.41 [95% CI, 2.57–7.56], *P* < 0.0001, versus OR, 3.15 [95% CI, 2.09–4.77], *P* < 0.0001).

For the control group (<60 years), there was a trend toward lower mortality in patients with higher antibody levels, albeit not at a statistically significant level. Mortality rates in younger adults did not differ by vaccination status. Due to the low number of deaths in younger adults, these results need to be interpreted with care.

### Risk estimation and adjustment for potential confounders.

In order to assess the risk associated with lower anti–SARS-CoV-2 spike antibodies, we built multiple logistic regression models for all endpoints. We further calculated Cox proportional hazard models for the primary endpoint in-hospital mortality to provide a second measure of risk. To limit the influence of potential confounders, these models were then adjusted for the covariates age, BMI, SARS-CoV-2 variant, T2D, hypertension, coronary artery disease (CAD), heart failure, stroke/transient ischemic attack (TIA)/cerebrovascular disease (CVD), and renal disease.

**Figure 4:**
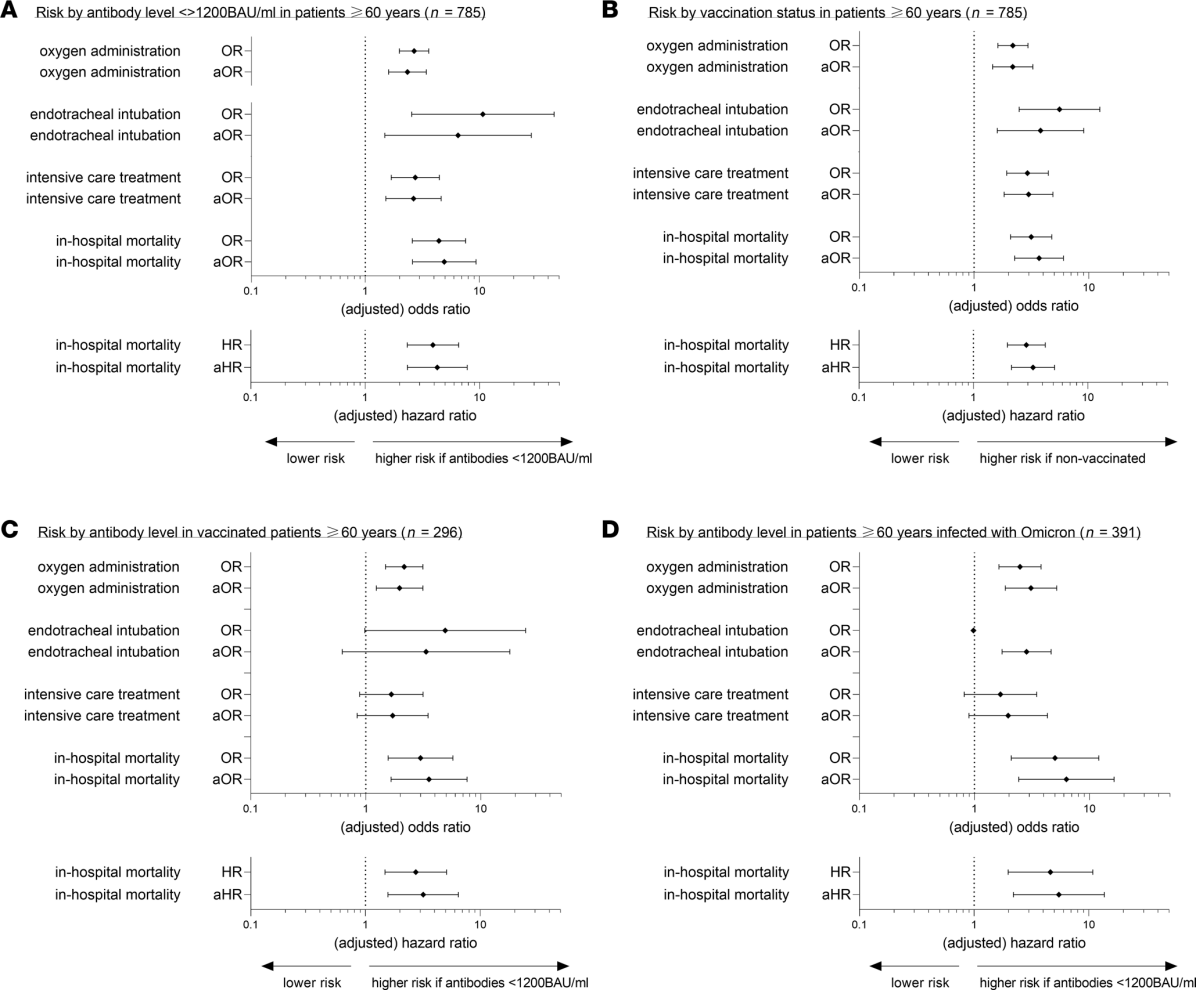
Risk of outcome in older adults. (**A**–**D**) Risk of outcome in older adults, aged 60 years or older, by antibody level above versus below 1,200 BAU/mL. (**A**) and vaccination status (**B**); risk of outcome by antibody level in vaccinated older adults (**C**) and in older adults infected with the Omicron variant (**D**). Unadjusted and adjusted odds ratios are shown for the outcomes oxygen administration, endotracheal intubation, intensive care admission, and in-hospital mortality. Unadjusted and adjusted hazard ratios are shown for in-hospital mortality. Adjusted odds and hazard ratios were calculated by multiple logistic and Cox regression analyses and adjusted for age, BMI, SARS-CoV-2 variant, type 2 diabetes, hypertension, CAD, heart failure, stroke/TIA/CVD, and renal disease.

Figure 4 shows risk of outcome by antibody level and vaccination status as both unadjusted and adjusted ORs for all endpoints and as hazard ratios for the primary endpoint in-hospital mortality. Results for vaccinated older adults and older adults infected with the Omicron variant are also presented. Unadjusted and adjusted risk ratios by antibody level and vaccination status for all endpoints are shown in Supplemental Figure 1 (supplemental material available online with this article; https://doi.org/10.1172/jci.insight.183913DS1).

After adjusting for potential confounders, older adults with antibody levels below 1,200 BAU/mL exhibited almost 5 times the mortality risk of patients above this threshold (adjusted OR [aOR], 4.92 [95% CI, 2.59–9.34], *P* < 0.0001). In addition, they were approximately 2.6 times more likely to be admitted to an intensive care unit (aOR, 2.64 [95% CI, 1.52–4.62], *P* = 0.00062). The odds for endotracheal intubation were 6.5 times higher and patients were more than twice as likely to require oxygen if antibody levels were below 1,200 BAU/mL (endotracheal intubation aOR, 6.50[ 95% CI, 1.48–28.47], *P* = 0.013; oxygen administration aOR, 2.34 [95% CI, 1.60–3.43], *P* < 0.0001).

In the Cox proportional hazards analysis, risk of death for older adults was more than 3 times higher if antibody levels were found to be lower than 1,200 BAU/mL (hazard ratio, 3.92 [95% CI, 2.34–6.56], *P* < 0.0001). Analogous to the logistic regression model, the results remained stable after adjusting for potential confounders (aHR 4.27 [95% CI, 2.34–7.81], *P* < 0.0001).

Older adults infected with the currently prevailing Omicron variant were more than 6 times more likely to die if antibody levels were below 1,200 BAU/mL (aOR, 6.31 [95% CI, 2.43–16.40], *P* = 0.00016).

In comparison with antibody levels below and above 1,200 BAU/mL, vaccination status was a weaker predictor of our primary endpoint, in-hospital mortality (aOR, 4.92 [95% CI, 2.59–9.34], *P* < 0.0001, vs. aOR, 3.68 [95% CI, 2.26–6.01], *P* < 0.0001).

### Mortality risk estimation by antibody titer increment.

In order to further quantify a possible dose-effect relationship between anti–SARS-CoV-2 spike antibodies and mortality risk in older adults, we calculated the increase in mortality risk with decreasing antibody levels in steps of 100 BAU/mL and 250 BAU/mL.

After adjusting for potential confounders, mortality risk increased by approximately 1.1 for each 100 BAU/mL decrease (aOR, 1.08 [95% CI, 1.05–1.11], *P* < 0.0001) and by 1.2 for each 250 BAU/mL decrease (aOR, 1.21 [95% CI, 1.13–1.30], *P* < 0.0001).

Results were comparable for older adults infected with the currently prevailing Omicron variant (100 BAU/mL steps: aOR, 1.08 [95% CI, 1.04–1.13], *P* = 0.00011; 250 BAU/mL steps: aOR, 1.22 [95% CI, 1.10–1.35], *P* = 0.00011).

## Discussion

### Key results.

In this prospective, multicenter cohort study on 785 older patients with COVID-19 and 367 controls, we were able to demonstrate for the first time to our knowledge that anti–SARS-CoV-2 antibody levels are highly predictive of outcome in this vulnerable patient group. Furthermore, antibody levels were a stronger predictor of in-hospital mortality than vaccination status.

Old age has been identified as a main risk factor for severe courses and COVID-19–related mortality (5, 6), and older adults remain vulnerable even with the currently prevailing, comparatively milder Omicron variant (6, 10). In order to ensure optimal protection of this important patient group, a correlate of protection is needed to identify patients at high risk of adverse outcomes, to assess the percentage of the population that is currently protected, and to guide the timing of future booster vaccinations.

Previous studies have shown that booster vaccinations lower the risk of reinfection or breakthrough infections, mitigate the severity of COVID-19, and lower mortality rates in older adults (33). However, given the interindividual variation and the reduced strength, quality, and durability of antibody responses associated with old age (13, 18, 20, 21, 23), generalized recommendations may be insufficient to protect individual patients.

To our knowledge, no data are currently available on the efficacy of antibody levels in this important patient subset, nor are there comparisons on the relevance of vaccination status versus antibody levels for predicting severe disease (33). Furthermore, no data are presently available to suggest a protective antibody threshold in older adults that may be used to assess the necessity of booster vaccinations.

In this study, we provide data on the clinical utility of measuring anti–SARS-CoV-2 spike antibodies and compare the relevance of antibody levels and vaccination status as a correlate of protection against adverse outcomes including COVID-19 mortality in hospitalized older adults with COVID-19.

### Strengths and limitations.

This study possesses multiple strengths. Firstly, this study has a high recruitment rate, which substantially reduces the risk of selection bias. Secondly, this study focuses on a hard primary endpoint, in-hospital mortality, which is independent of subjective clinical assessment and thus minimizes the risk of assessment bias associated with softer clinical endpoints (34). While our secondary endpoints are more susceptible to this type of bias, they nonetheless serve to complement our results with regard to less severe cases.

Thirdly, the anti–SARS-CoV-2 antibody test used in this study is widely available, has short turn-around times, and has been shown to have a high sensitivity for detecting neutralizing antibodies against SARS-CoV-2 and to maintain that sensitivity over time (35). Fourthly, all regression models conducted in this study were adjusted for various potential confounders that have been identified as significant risk factors for more severe courses of COVID-19, including age, BMI, SARS-CoV-2 variant, T2D, hypertension, CAD, heart failure, stroke/TIA/CVD, and renal disease (6, 11, 12, 36, 37).

With regard to limitations, it should be noted that this study investigated hospitalized patients, and this fact may limit the generalizability of its findings to outpatient settings. However, since severe cases of COVID-19 predominantly necessitate hospitalization, we considered it essential to focus on this patient group. While we adjusted for several confounders that have been linked to severe courses, the observational nature of this study carries the risk of unmeasured confounding that may have introduced bias. Since hazard ratios are estimated conditional on survival, hazard ratios are subject to built-in selection bias and need to be interpreted with care.

Of note, endotracheal intubation was only performed in 33 older patients. While the results for this secondary endpoint were still statistically significant, the low patient count may have affected results.

It should also be noted that only anti–SARS-CoV-2 spike antibody levels were measured and no direct virus neutralization test (VNT) was conducted. However, in a comparison of different antibody assays (38), the qualitative agreement between the Roche assay employed in this study and a VNT was 97.6%. Of the antibody assays examined, only the Roche Elecsys anti–SARS-CoV-2 S exhibited a near constant sensitivity over the study duration of 12 months. Spike protein–specific antibody tests showed good correlation to a VNT over the study duration (Spearman’s rank [rS], 0.74–0.92), with the antibody level at 3 months being the best predictor of VNT 12 months after disease onset. Correlation between antibody levels and virus neutralization titers was comparable for all spike protein–specific assays, with 100 BAU/mL and 1,000 BAU/mL after 100 days leading to a mean virus neutralization titer of 8–16 and 64, respectively after 12 months (38).

In order to improve comparability between different antibody assay platforms, the WHO introduced an international standard for anti–SARS-CoV-2 immunoglobulin derived from pooled human plasma of 11 patients convalescing from SARS-CoV-2. With the introduction of this standard, interlaboratory variation was reduced more than 50 times for neutralization and more than 2,000 times for ELISA (39). However, contrary to the original intention of introducing this unit, significant differences have been observed between assays calibrated against this standard, and test results have not yet been completely harmonized (38). While there are a number of comparative studies between the most common test platforms (38), this variability needs to be taken into account in applying our results to other platforms.

This study did not address cell-mediated immunity and therefore did not evaluate the participants’ immune status. Viral clearance necessitates both humoral and cellular immune responses. T cell–mediated immunity is essential for identifying SARS-CoV-2 variants, eliminating SARS-CoV-2, and establishing durable long-term memory responses (40). Furthermore, T cell responses may confer some protection in patients with poor antibody responses (40). The strength of the antibody response over the course of COVID-19 increases with increasing disease severity (41). A similar correlation has been shown for T cell responses, most notably for CD8^+^ T cells (40). However, in contrast to antibody levels, measures of cellular immune responses are less widely available in routine clinical laboratories, carry higher costs, and are less established as correlates of immunity in clinical practice (42). Nonetheless, future studies are warranted to elucidate the interplay between T cell levels, antibody titers at the onset of the infection, and outcome in COVID-19.

### Interpretation.

Both reduced immunogenicity and reduced durability of vaccine-induced immune responses were reported in older adults (13). However, a large nationwide study from Israel analyzing data from more than 1 million participants stated that booster vaccinations against COVID-19 significantly increased protection against severe illness in adults aged 60 years or older (43). Timely booster vaccinations are therefore particularly important in older adults.

The worse outcomes observed in older adults have previously been attributed to an accumulation of comorbidities and progressive frailty (11). Accordingly, the average number of comorbidities was significantly higher in older versus younger adults and increased with each decade in older adults. However, the number of comorbidities did not reach statistical significance in multiple logistic regression analysis, and adding this variable did not affect the statistical significance of our results.

Since the onset of the pandemic, the seroprevalence of anti–SARS-CoV-2 antibodies has increased considerably due to preceding infections, vaccinations, or varying combinations thereof. Recurrent exposure from reinfections or booster vaccinations has been shown to confer added protection against COVID-19 severity (31, 33).

However, antibody responses vary widely between individuals, are weaker and less effective with age, and decline more quickly in older adults. In particular, those with the highest risk of adverse outcomes may therefore not have sufficiently high antibody levels to ensure the best possible protection. Measuring antibody levels in older adults and providing booster vaccinations if antibody levels are low may therefore be of use in protecting these patients. In addition, measuring antibody levels at hospital admission of older adults may be helpful in identifying high-risk individuals who would benefit from intensified treatment regimes.

While COVID-19–related hospitalization and mortality rates are lower under the currently prevailing Omicron variant, older adults and patients with severe comorbidities that are more common in older adults account for the majority of deaths (6, 10). In addition, given that the virus persists in the human population (1) and the reduction in both testing and detailed reporting of test results (3), there may be a considerable number of unreported and undiagnosed cases.

With regard to emerging SARS-CoV-2 variants, the loss in efficiency of antibodies formed against previous variants or against vaccines that have not been updated is also a considerable concern.

For the Omicron variant, alterations of the spike protein that are associated with an extensive evasion of neutralizing antibodies have been described (44). Nonetheless, sufficiently high antibody titers against preceding variants were still observed to provide protection. This is in accordance with our study, as adjusting for virus variant did not affect the statistical significance of our results.

Nonetheless, in case of a substantial loss in antibody efficiency with the emergence of a new variant, future studies are needed to ascertain if higher antibody thresholds are required to protect vulnerable patient groups.

Future studies are also required to investigate whether preformed antibody levels at the onset of a SARS-CoV-2 infection indicate protection against long-COVID-19.

The antibody level present on hospital admission is derived from both previously formed antibodies and antibodies formed in response to the current infection. Therefore, prolonged infections preceding hospital admission may limit the prognostic utility of antibody levels on hospital admission. In our data, median time from symptoms to hospitalization was 3 days (IQR, 1–7). Despite this limitation, older adults with antibody levels below 1,200 BAU/mL on hospital admission are expected to have had similar or slightly lower antibody levels at the time of exposure. Maintaining antibody levels > 1,200 BAU/mL in older adults may therefore be argued to be a conservative target.

It should be noted that vaccination status was a weaker predictor of mortality than antibody levels. As antibody levels have been shown to decrease substantially over time (20), the relevance of being vaccinated is likely to decrease with increasing time from the last dose. This suggests that monitoring antibody levels constitutes a better and more direct approach for safeguarding older adults from adverse COVID-19 outcomes.

While we did observe significantly poorer outcomes in older adults below 1,200 BAU/mL of anti–SARS-CoV-2 spike antibodies, this value needs to be verified in separate cohorts. Nevertheless, the high interindividual variability in antibody responses to vaccination, combined with the reduced antibody response and lower durability of immune responses following vaccination in older patients, highlight the importance of timely administration of booster doses.

In order to translate this study’s findings to clinical practice, future studies are warranted to compare COVID-19 outcomes of older adults following personalized, antibody level–guided vaccination regimes with patients following standard recommendations for vaccination.

### Conclusion.

In older adults, antibody levels were a stronger predictor of in-hospital mortality than vaccination status. This suggests that monitoring antibody levels constitutes a better and more direct approach for safeguarding older adults from adverse COVID-19 outcomes.

## Methods

### Sex as a biological variable.

Our study examined male and female participants, and findings were similar for both sexes.

### Study design and participants.

We conducted a prospective, multicenter cohort study involving hospitalized patients from 5 hospitals in Austria. The recruitment period spanned from August 1, 2021, to April 10, 2022.

Patients were considered eligible to participate in the study if a positive SARS-CoV-2 test result from a PCR-based assay had been obtained and a blood sample had been procured at the time of hospital admission. Patients were excluded if they had been hospitalized previously during the study period or if they had not yet been discharged at the conclusion of the study.

Sample size calculation was conducted for cohort studies with dichotomous outcomes and independent proportions (45, 46). Since there were no previous studies examining mortality rates by high and low antibody levels in older adults (33), we estimated a mortality rate of 20% in patients with low antibody levels and 10% in patients with high antibody levels based on the prevailing mortality rates at the commencement of the study (6). Type I error rate α (2-sided significance level) was set at 0.05, power (1-β) at 0.8, and the expected dropout rate was set at 10%. The ratio between number of patients in group 1 (low antibody levels) and group 2 (high antibody levels) was set to 1. Without the correction for continuity (45), a minimum total sample size of 420 participants was required. Employing the correction for continuity, the sample size calculation indicated a minimum sample size of 243 patients per group and 486 patients in total.

### Variables.

The primary endpoint of this investigation was defined as in-hospital mortality from any cause.

Secondary outcomes encompassed admission to an intensive care unit, need for endotracheal intubation, and oxygen administration.

Patients aged 60 years or older were categorized as older adults. Patients who had received either 1 dose of an accepted single-dose vaccine or completed 2 doses of an accepted 2-dose series against SARS-CoV-2 were classified as vaccinated.

Predefined covariates were chosen based on established risk factors with an increased likelihood of severe disease and higher mortality in COVID-19. Even among older patients, age constitutes one of the main risk factors in COVID-19, presumably due to a progressive accumulation of comorbidities and frailty (11). Various comorbidities including T2D, obesity, renal diseases, and cardiovascular diseases such as hypertension, CAD, and heart failure have also been identified as risk factors in COVID-19 and were, thus, included in the analysis (12, 36). In addition, mortality rates and disease severity is known to vary by SARS-CoV-2 variant, with the Delta variant being associated with higher mortality rates compared with the Omicron variant (6).

### Data sources and measurements.

Anti–SARS-CoV-2 spike antibodies were measured at the Central Medical Laboratories in Feldkirch, Austria, on Roche Cobas 6000 or Cobas 8000 systems, utilizing the Elecsys Anti–SARS-CoV-2 S assay for quantitative detection of total antibodies against the receptor binding domain (RBD) of the WA1 SARS-CoV-2 spike protein.

Clinical data were collected from patient records. Between August 2021 and December 2021, SARS-CoV-2 variants were identified by genetic sequencing. Between January and April 2022, epidemiological data indicate that the Omicron variant had superseded the previously circulating variants (47). Hence, patients who were admitted with a positive PCR-based test result for SARS-CoV-2 during this time frame were categorized as Omicron positive.

### Statistics.

All statistical analyses were performed using the IBM Statistical Package for the Social Sciences (SPSS), version 29. To assess statistical significance, we employed Mann-Whitney *U* tests or Kruskal Wallis tests for continuous and χ^2^ tests for categorical variables. A 2-sided *P* < 0.05 was regarded as statistically significant.

In order to evaluate the risk associated with lower anti–SARS-CoV-2 spike antibody levels, we built logistic regression models for each endpoint using a threshold of 1,200 BAU/mL. This threshold was obtained by dividing the measuring range in half to generate low and high categories (31). Regression models were constructed through a direct model-building approach, where all independent variables were entered simultaneously. Primary and secondary endpoints were entered as dichotomous dependent variables, while predefined covariates were input as independent variables. ORs were presented with 95% CI.

As an additional risk assessment method, we built Cox proportional hazards models to determine hazard ratios for our primary outcome measure, in-hospital mortality. The proportional hazard model was constructed following the approach described in the preceding paragraph. Time to event was entered in days measured from hospital admission. The proportional hazards assumption was confirmed by testing for interactions with time using Cox regression with time-dependent variables. Linearity for quantitative predictors was assessed by plotting Martingale residuals against continuous covariates (48). Kaplan-Meier curves were used to depict cumulative survival over time. Statistical significance of differences in survival over time was assessed using Log-rank (Mantel-Cox) tests. Survival analysis was conducted from hospital admission with patients being followed until 90 days after admission.

Next, both logistic regression and Cox regression models were adjusted for potential confounders that were selected based on the modified disjunctive cause criterion (49). These confounders included age, BMI, SARS-CoV-2 variant, T2D, hypertension, CAD, heart failure, stroke/TIA/CVD, and renal disease. With regard to the secondary endpoint endotracheal intubation, the number of covariates had to be limited due to the relatively low number of events to avoid overfitting of the regression models. We therefore incorporated the confounders that showed the highest potential influence on outcome in previous studies (age, BMI, and SARS-CoV-2-variant) for this endpoint (6, 12, 36).

The robustness of our models was then verified by rebuilding all regression models while executing bootstrapping with 2,000 samples. Finally, we used Hosmer-Lemeshow tests to confirm goodness of fit.

### Study approval.

The local IRB, Ethikkommission Vorarlberg, Roemerstrasse 15, A-6901 Bregenz, approved the study and waived the need to obtain informed consent from the study participants due to the observational nature of this study. The study was carried out in accordance with the Declaration of Helsinki of 1975 (revised 2013) and Good Clinical Research Practice.

### Data availability statement.

As personal individual information is included in the dataset, the data pertaining to this investigation is not publicly available to protect study participant privacy. However, an anonymized version will be shared upon reasonable request to the corresponding author. Values for all data points in graphs are reported in the Supporting Data Values file.

## Author contributions

PF and SM were responsible for conceptualization, methodology, investigation, and data curation. SM conducted the formal analysis with input and mentorship of PF, WH, JC, and CHS. PF, SM, PR, CHS, AL, and HD were responsible for data analysis and interpretation. Resources were provided by PF, HD, and MF. The original draft was drawn up by SM. The data were verified by SM, PR, and PF. Critical review and editing of the manuscript was performed by all authors. All authors had full access to the underlying data. All authors contributed important intellectual content during manuscript drafting and or revision, and accept accountability for the overall work. All authors have accepted responsibility for the entire content of this manuscript and approved its submission.

## Supplementary Material

Supplemental data

ICMJE disclosure forms

Supporting data values

## Figures and Tables

**Table 1:**
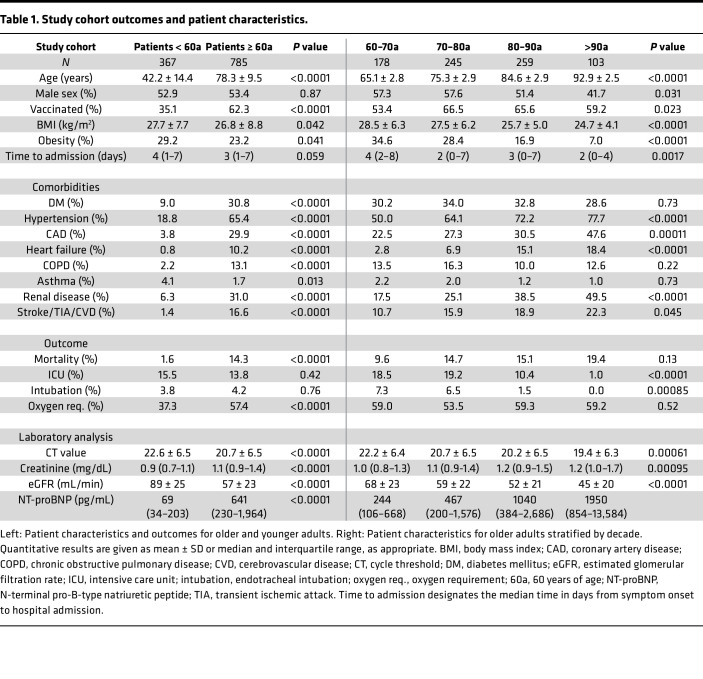
Study cohort outcomes and patient characteristics.

**Table 2:**
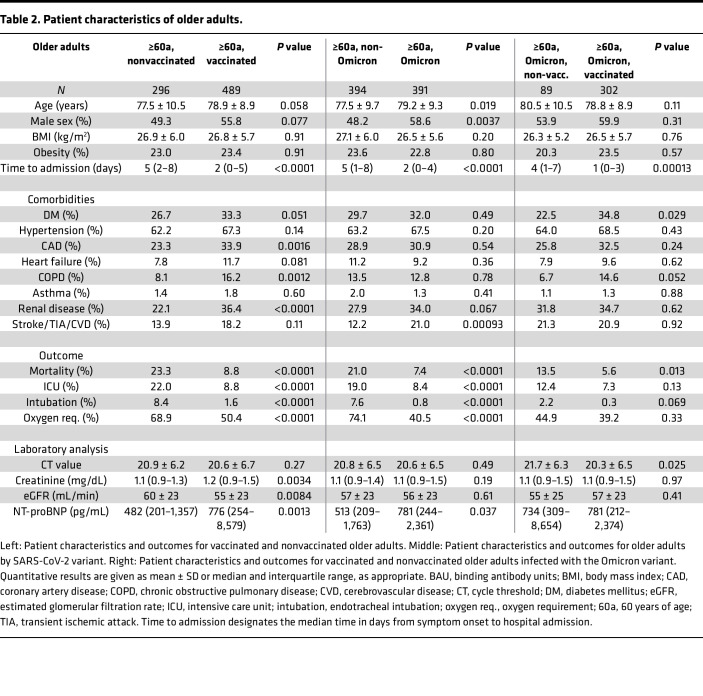
Patient characteristics of older adults.

**Table 3:**
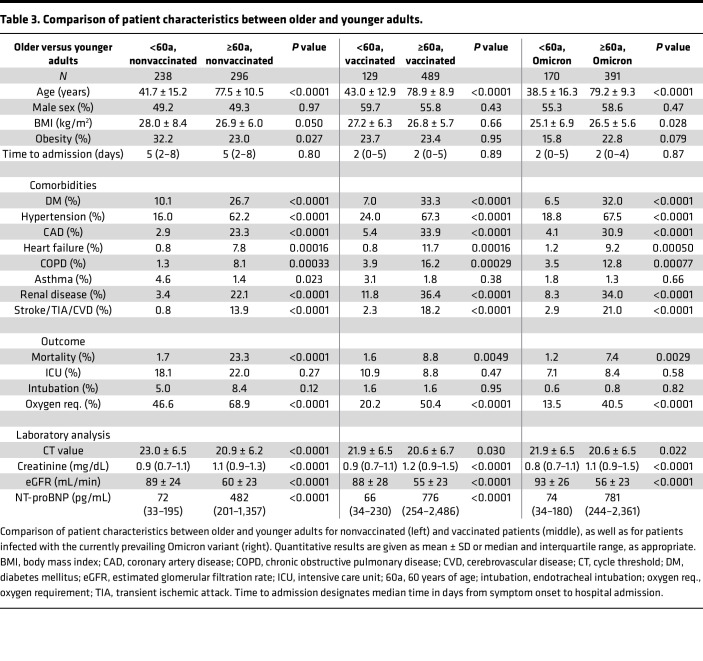
Comparison of patient characteristics between older and younger adults.

## Version 1 10/22/2024

Electronic publication
