# Supplementary material for: Antibody levels versus vaccination status in the outcome of older adults with COVID-19
Source: JCI Insight. 2024 Oct 22;9(20):e183913. doi: 10.1172/jci.insight.183913 (PMC11529978; doi:10.1172/jci.insight.183913)
Supplement: Supplemental data [file jciinsight-9-183913-s084.pdf]

Supplementary material to

Evaluation of antibody levels versus vaccination status in the outcome of older adults with COVID-19: a prospective cohort study

Table of contents

Supplementary figure 1 page 2

Risk of outcome in risk ratios

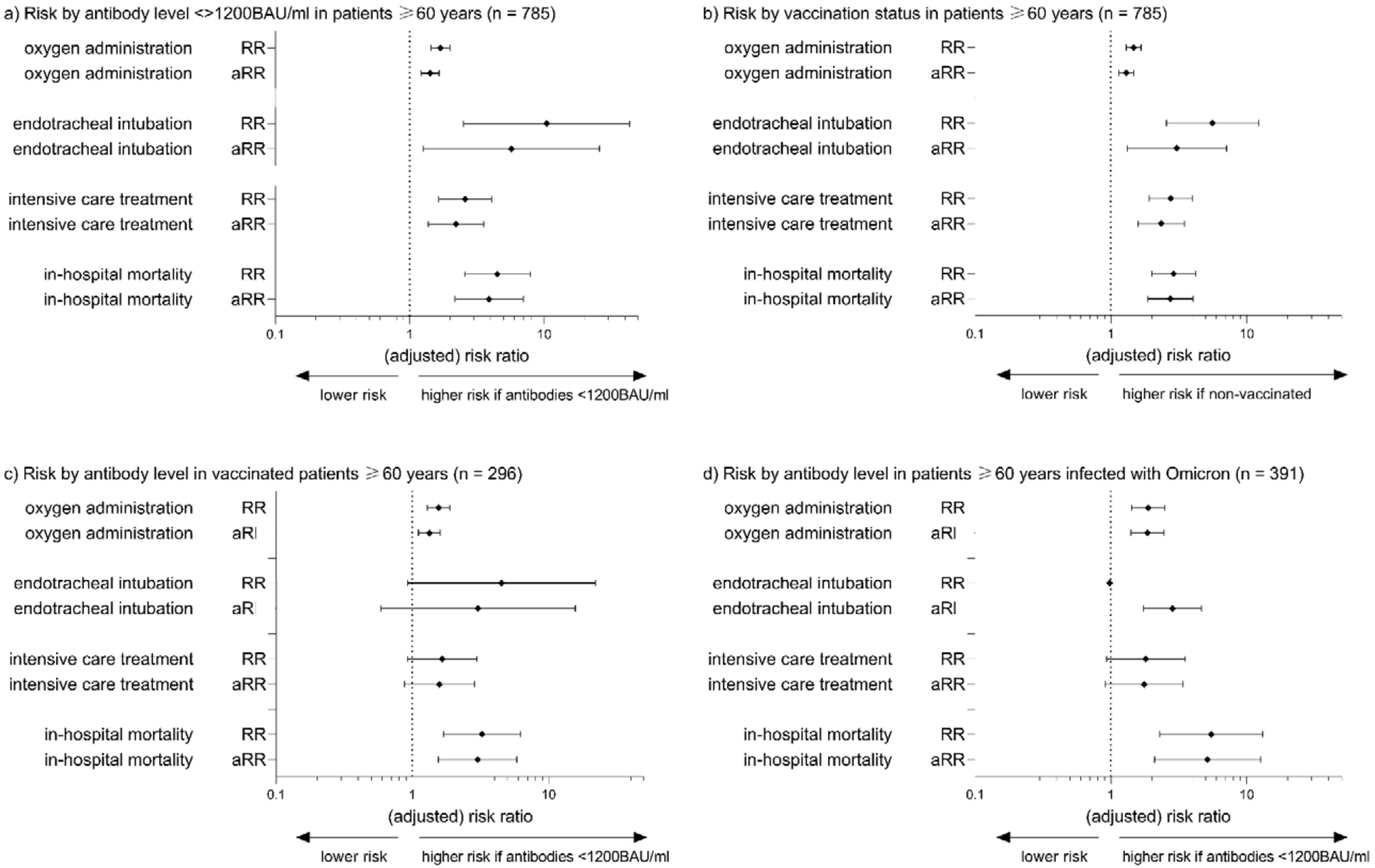

Supplementary figure 1. Risk of outcome in older adults, aged 60 years or above, by antibody level above vs. below 1200BAU/ml (a) and vaccination status (b); risk of outcome by antibody level above vs. below 1200BAU/ml in vaccinated older adults (c) and in older adults infected with the Omicron variant(d). Unadjusted and adjusted risk ratios (RR) are shown for the outcomes oxygen administration, endotracheal intubation, intensive care admission, and in-hospital mortality. Risk ratios were calculated using modified Poisson regression models. Adjusted risk ratios were adjusted for age, BMI, SARS-CoV-2 variant, type 2 diabetes, hypertension, CAD, heart failure, stroke/TIA/CVD, and renal disease.
